# #Scamdemic, #Plandemic, or #Scaredemic: What Parler Social Media Platform Tells Us about COVID-19 Vaccine

**DOI:** 10.3390/vaccines9050421

**Published:** 2021-04-22

**Authors:** Annalise Baines, Muhammad Ittefaq, Mauryne Abwao

**Affiliations:** William Allen White School of Journalism & Mass Communications, University of Kansas, Lawrence, KS 66045, USA; muhammadittefaq@ku.edu (M.I.); mauryneabwao@ku.edu (M.A.)

**Keywords:** COVID-19 vaccine, echo chamber, online discussions, misinformation, Parler, social media

## Abstract

This study aims to understand public discussions regarding COVID-19 vaccine on Parler, a newer social media platform that recently gained in popularity. Through analyzing a random sample (*n* = 400) of Parler posts using the hashtags #COVID19Vaccine and #NoCovidVaccine, we use the concept of echo chambers to understand users’ discussions through a text analytics approach. Thematic analysis reveals five key themes: reasons to refuse the COVID-19 vaccine (40%), side effects of the COVID-19 vaccine (28%), population control through the COVID-19 vaccine (23%), children getting vaccinated without parental consent (5%), and comparison of other health issues with COVID-19 (2%). Textual analysis shows that the most frequently used words in the corpus were: nocovidvaccine (348); vaccine (264); covid (184); covid19 (157); and vaccines (128). These findings suggest that users adopted different terms and hashtags to express their beliefs regarding the COVID-19 vaccine. Further, findings revealed that users used certain hashtags such as “echo” to encourage like-minded people to reinforce their existing beliefs on COVID-19 vaccine efficacy and vaccine acceptance. These findings have implications for public health communication in attempts to correct false narratives on social media platforms. Through widely sharing the scientific findings of COVID-19 vaccine-related studies can help individuals understand the COVID-19 vaccines efficacy accurately.

## 1. Background and Context

After major media outlets in the United States such as CNN, Fox News, and CNBC, predicted that Joe Biden would win the 2020 presidential election on 7 November 2020, several right-wing media outlets started to spread claims that the election was rigged, and President Donald Trump should have won the 2020 U.S. presidential election [1]. Social media users who believe the election was rigged supported such claims through sharing misleading content about election results. During this time, the United States was making preparations to being distributing the first of several COVID-19 vaccines after President Trump claimed the vaccine would be available before the election was decided [2]. Spurred by this timing, Trump supporters on social media questioned the efficacy and safety of the COVID-19 vaccine, particularly on Parler, which became one of the most downloaded social media applications on the Apple store [3]. In the time between the close of ballot collection on 3 November 2020 and the announcement by new media that Biden was projected to win, Parler had been downloaded almost one million times [4]. Unlike other social media platforms, Parler branded itself as a “non-biased, free speech social-media platform.” In particular, it noted the site does not include fact checkers [5].

Parler received more interest after conservative pundits and right-wing agitators joined the platform and began to spread false theories and misinformation related to the election and the COVID-19 vaccine [6]. Influential individuals and opinion leaders such as American Senators Ted Cruz and Rand Paul have praised the platform for its free speech component. In the wake of the deadly U.S. Capitol riots on 6 January 2021 Amazon, Google, and other major corporations removed the platform from their app stores and Parler was eventually deplatformed by the hosting provider Amazon Web Services [7]. After that, Parler users shifted to other social media platforms such as Rumble and Gab [8]. The platform returned online on 15 February 2021 [9]. 

With the proliferation of user-generated content on social media, this study aims to understand public discussions concerning the COVID-19 vaccine on Parler. Understanding the role of new social media platforms in vaccine communication provides an opportunity for scholars and practitioners to contextualize the misinformation and conspiracy theories related to vaccines in future pandemics. Previously, several studies have examined the role of social media platforms in regard to the anti-vaccination movement [10]. For example, Bonnevie et al. [11] studied vaccine opposition on Twitter and concluded that the platform is being used to increase mistrust in health authorities, which could impact larger populations in regard to vaccinations in future pandemics. Similarly, Basch et al. [12] explored vaccine communication in YouTube videos and found that the majority of the videos mentioned COVID-19 vaccine manufacturing processes. These findings imply speculations and doubts about vaccine readiness, which ultimately can lead individuals to distrust vaccine development. Yet, as newer social media sites emerge, they provide an opportunity to expand this work [13]. Little attention has been paid to newer social media platforms such as Parler to make sense of user discussions about COVID-19 vaccines. To understand user discussions, we situate our study within the echo chamber conceptual framework and investigate whether echo chambers exist within Parler. Methodologically, we use a text analytics approach (thematic and textual) to contribute to the growing literature on vaccine hesitancy, explore the role of new social media platforms in creating echo chambers, and delve into COVID-19 vaccine efficacy debates. 

### 1.1. Echo Chambers and Social Media Platforms

Echo chambers often appear on social media platforms where individuals gather and are surrounded by like-minded people in terms of political and ideological orientation [14,15]. These platforms allow individuals more control over their information exposure, increase opinion-reinforcing information, and create polarization on certain health topics such as vaccines [16]. For example, Schmidt et al. [17] conducted a study on vaccine hesitancy content on Facebook and found the existence of echo chambers on the platform, in which pro- and anti-vaccination attitudes polarize the users. The study also found that users from the anti-vaccination community consume more sources compared to the pro-vaccine users, which is consistent with results from previous studies [18]. Further, while both narratives gained attention on Facebook over time, anti-vaccine pages displayed more cohesive growth (i.e., pages liked by the same people), while the pro-vaccine page showed growth in a more highly fragmented fashion (i.e., pages liked by different people). 

Echo chambers have recently come under scrutiny because they enable individuals to promote and facilitate conspiracy theories and misinformation which leads to skewed evaluation of objective facts and polarized opinion on controversial topics [13,17,19]. When individuals join radical groups on the internet, they have easy access to and frequently use social media platforms as echo chambers as a way to voice their opinions on controversial topics [13]. Studies have shown that these homogenous subnetworks reinforce bias through cognitive dissonance [20]. Echo chambers are also seen as drivers of polarization, especially with unreliable anti-vaccine content, because large numbers of internet users seek their health information from unreliable sources circulating in these chambers [21,22]. Besides facilitating the spread of political extremism [23], echo chambers have also been shown to disperse misinformation about infectious diseases [24].

Networked communities provide opportunities to study and understand online discussions on vaccine hesitancy and its implications for society and science communities [25]. Over the last two decades, researchers found that scientific results may lead to confusion over health information regarding vaccines if they are not explained in simple terms for individuals lacking a medical or scientific background [26,27,28,29]. The spread of the interpreted information using social media platforms yields considerable influence over the general public, specifically when it comes to making decisions on whether to vaccinate. The role of social media platforms and echo chambers in shaping perceptions and amplifying anti-vaccination messaging, as is the case with the COVID-19 vaccine, cannot be ignored [28], and little research has examined vaccination discussions in echo chambers. To the best of our knowledge, there are no empirical studies that focus on COVID-19 vaccination discussions on social media platforms such as Parler.

Three key considerations should be taken into account when using echo chambers as conceptual framework: networks are homogenous, topics are controversial, and political predispositions are strong [13]. We argue that COVID-19 vaccines were being publicized close to and during the U.S. presidential election period in 2020. The Parler network became a popular forum among right-wing individuals to gather and create a homogenous group to spread conspiracy theories and misinformation concerning the COVID-19 vaccine [30]. Hence, we argue that this socio-political scenario makes an ideal case to apply the echo chamber conceptual framework to understand COVID-19 vaccine conversations on Parler. 

### 1.2. Vaccine Misinformation on Social Media Platforms

Historically, discussions concerning the topic of vaccines has generated false and misleading claims, fake content, and conspiracy theories across the globe [31,32,33]. However, contemporary scholarship has demonstrated that misinformation and conspiracy theories can spread much faster on social media compared to mainstream media and these conspiracy theories have influenced the way people think about vaccinations leading the public to question the need for immunization [34]. A growing body of research shows that consumers struggle to evaluate the credibility and accuracy of online content, especially regarding health issues. For example, several researchers found in their experimental studies that exposure to online information critically examining vaccinations leads to stronger anti-vaccine beliefs, due to individuals not taking the credibility of the content into account [35,36,37,38].

Extensive work has been done to document conspiracy theories and misinformation on COVID-19 [39,40,41,42]; however, there is a gap in the research on COVID-19 vaccine information circulating on newly developed social platforms such as Parler. Research indicates that false information about health issues on social media spreads faster than accurate information [43]. Federal institutions such as the Centers for Disease Control and Prevention (CDC) and local and state health departments disseminate the most accurate information on health issues as it is known at the time [44]; however, they face numerous challenges in combating with health misinformation. Thus, understanding online users’ discussion patterns on social media platforms such as Parler, which recently gained popularity among users but have yet to receive scholarly attention is important to help design communication strategies for policy makers in health communication.

### 1.3. Previous Research on Parler

Parler identifies itself as a “free speech” site and differs from other social media platforms such as Twitter, Facebook, YouTube, and Gab because it offers an additional extensive set of self-served moderation tools. For instance, Parler allows its users to tip each other by sending small amounts of money for content they produce on the platform. Such an incentive can motivate users to produce and share content on the platform, unlike other sites such as YouTube, where content creators have to gain a certain number of subscribers to get monetized [8]. These affordances can also motivate users to spread false claims, misleading statements, and conspiracy theories on current topics like the COVID-19 vaccine in search of more reach and more tips. 

Recently, a few studies analyzed Parler content to make sense of “parleys”—the content posted, shared, and commented on by Parler users—in various contexts. Aliapoulios et al. [8] examined 13 million users’ information and 180 million parleys. They showed that Parler gained followers shortly after real-world events related to online censorship on mainstream platforms such as Twitter and events surrounding U.S. politics. Further, users mainly share content related to U.S. politics, specifically in support of Donald Trump and his efforts during the 2020 U.S. elections, as well as conspiracy theories generated by groups such as QAnon. Another study by Munn [45] examined 350,000 parleys shortly before and during the 6 January 2021 U.S. Capitol riots. The study conceptualized Parler as a preparatory media that is used for incitement, legitimating, and mobilizing users. Preparatory media plays an active role in framing events, identifying target audiences, setting agendas, and ensuring that all ideas shared on the platform are not divergent from the primary goal [45]. While the findings of these studies reveal that mainstream and new social media platforms easily spread misinformation and conspiracy theories around socio-political and health issues, to the researchers’ knowledge, our study is the first to examine parleys to understand COVID-19 vaccine discussions.

### 1.4. Aims of the Study

This study aims to explore how individuals discuss and respond to the COVID-19 vaccine on the platform Parler during the early news of the novel coronavirus vaccine rollout. Further, we unearth major themes of conversation related to the COVID-19 vaccine. Following Geiß et al. [13] approach and taking three key considerations into account (networks are homogenous, topics are controversial, and political predispositions are strong), we examine whether echo chambers exist on Parler related to the topic on COVID-19 vaccine. More specifically, we pose the following research questions in this study: 

RQ_1_: What are the major themes in users’ discussions related to COVID-19 vaccines on Parler?

RQ_2_: To what extent do echo chambers exist on the Parler platform to reinforce misinformation about COVID-19 vaccines?

## 2. Methods

### 2.1. Data Collection and Sampling

With the help of key words and hashtags such as #covid19, #COVID19Vaccine, #NoCovidVaccine, #coronavirus, #pandemic, #nopandemic, #plandemic, #scaredemic, #scamdemic, #china, #trump, #fakenews, #billgates, #maga, #faucci, and #notovaccines, we found a total of more than 7000 parleys about the COVID-19 vaccine. These hashtags were chosen based on the trends during the selected timeframe of the study. Our search strategy comprised of two steps: (1) initially, we collected all parleys based on the above-mentioned hashtags and key words, and (2) after initial screening, we narrowed down our search to the two most commonly used hashtags #COVID19Vaccine and #NoCovidVaccine. Our screening was based on the relevancy of the content related to COVID-19 vaccine. For instance, many of the hashtags that mentioned Bill Gates, MAGA, and fake news were not related to the COVID-19 vaccine. However, users who included these terms in parleys were discussing the 2020 presidential election, general politics, and Bill Gates’ purported agenda to dominate the world. Therefore, our final inclusion of parleys only consisted of two hashtags (#COVID19Vaccine and #NoCovidVaccine). It is important to note that these two hashtags were present in each parley and our search strategy showed that these two hashtags were the most commonly used to post content related to COVID-19 vaccine. To acknowledge the limitations of using two hashtags, we cannot infer that #COVID19 Vaccine was used to advocate or reject vaccine acceptance and #NoCovidVaccine was used to increase or decrease vaccine hesitancy without reading the contents of the parley. Keeping these limitations in mind, we analyzed our data by combining both hashtags to make sense of discussions regarding COVID-19 vaccine on Parler. A random sample (*n* = 400) of parleys were manually collected between 20 November 2020, and 6 January 2021, (before a temporal shut down of the platform) from this larger sample (*n* = 7000). Captions and affiliated indicia—symbols, signs, and distinguishing hashtags—were examined for this study. The average number of words in each parley was 26.2.

### 2.2. Thematic Analysis 

The qualitative data were analyzed first using Braun and Clarke’s [46] thematic analysis to identify, analyze, and report themes within the data. This inductive approach is ideal for organizing, describing, and interpreting the qualitative data [46]. After data was collected, cleaned, and organized in Microsoft Excel, all authors familiarized themselves with the complete dataset. Then, the authors used an open-coding approach to organize the data by reading each parley line by line including hashtags. This coding process has been used previously to understand textual data from the internet and social media platforms [34]. During the first phase, all three authors coded openly for potential themes. The coding sheet was constantly updated as each researcher coded the parleys for themes. In the second phase, two authors used a focused coding approach to understand the most frequently occurring themes [47]. In the final phase, the themes were further refined, modified, and merged following further discussion and agreement among the research team. Two authors together removed inconsistencies based on the coding and discussion. Further, the authors consulted a qualitative researcher in the authors’ institution to achieve an accepted level of informal reliability and validity. Our study followed Lincoln and Guba’s [48] approach which suggests using four definitive criteria (e.g., credibility, dependability, confirmability, and transferability) to ensure trustworthiness and acceptability of qualitative data (see also Ahmadian et al. [49]). Finally, illustrative names (e.g., population control through COVID-19 vaccine) were given to the themes [50]. After coding and discussing among the research team, five themes emerged from the dataset. The unit of analysis for this study was each parley regardless of the number of words and hashtags.

### 2.3. Textual Analysis

To achieve the aims of the study, we then used an automated text analytics approach to extract, analyze, unearth, and visualize insights in a corpus of text-based comments (see Khan et al. [51] for text-analytics method). Voyant-tools (http://voyant-tools.org/ accessed on 16 April 2021), a free, web-based text analytics tool and Microsoft Excel helped us achieve this aim. Voyant-tools was developed by Stéfan Sinclair and Geoffrey Rockwell for textual analysis and visualization in the field of social sciences and humanities [52]. The tool allows researchers to perform different types of analyses such as word cloud, exhibiting trends in the text file, and providing summaries and links between terms. We first sifted parleys to find the most commonly used terms and created word clouds related to the searched hashtags. 

## 3. Findings

This study aimed to analyze parleys using a text analytics approach to understand Parler users’ discussions around COVID-19 vaccines and a thematic analysis to identify common themes within these discussions which answers RQ1. Our work is guided by the echo chamber conceptual framework to understand to what extent echo chambers exist on the platform. This study is possibly the first in health communication to investigate parleys and user’s online discussions regarding the COVID-19 vaccine. Our analysis revealed five major themes: (a) reasons to refuse the COVID-19 vaccine (40%), (b) side effects of the COVID-19 vaccine (28%), (c) population control through the COVID-19 vaccine (23%), (d) children getting vaccinated without parental consent (5%), and (e) comparison of the mortality of other diseases with that of COVID-19 (2%). Many of the statements included external links to websites that were not credible, such as humansarefree.com, welovetrump.com and nationalfile.com. Further, our findings show that misinformation about the efficacy of the COVID-19 vaccine was prevalent on the platform and users disseminated these false claims using several hashtags. Following Geiß et al.’s [13] approach, our findings show that the parleys were homogenous (i.e., none of the Parler users support the COVID-19 vaccine), the topic was controversial (i.e., debate on getting vaccination against COVID-19), and Parler users created echo chambers within the platform to discuss the COVID-19 vaccine as a controversial and politically charged topic. Below we provide an overview of the five themes and corresponding parley examples.

### 3.1. Reasons to Refuse COVID-19 Vaccine

A majority of Parler users provide several reasons for refusing the COVID-19 vaccine. Not only do they share posts stating that they refuse to receive the COVID-19 vaccine, but also share posts of healthcare workers hesitating to receive the vaccine. For example: 

In Europe, a huge percentage of the population (around 50% or more depending on the country) are hesitant to receive the vaccine. In the U.S., polls show that at least 30% of the population will refuse outright, while 60% of people are hesitant about effectiveness. Even large numbers of healthcare workers are refusing the vaccine, and these are the people with the most pressure to submit or face consequences #endlockdownsnow #masksdontwork #nocovidvaccine #scamdemic (31 December 2020). 

This example shows that users make claims with statistics from different countries to justify their refusal in taking the COVID-19 vaccine but do not provide any scientific or nonscientific sources such as an empirical studies or mainstream media news. To make the claims credible, users mention that healthcare workers are among those who refuse to get vaccinated. These findings show that users use echo chambers to support their refusal with claims from government officials refusing the vaccine. Newer vaccines can cause backlash among communities and create an ambiguity aversion meaning that people are influenced by the unknown risks that may stem from more side-effects of the vaccine than the associated risks of the disease.

Some users strongly advocate by using hashtags that people should not get vaccinated, or governments should not force people to receive the COVID-19 vaccines. For instance, one user wrote: 

No way should anyone get this vaccine. If a libnut wants it then great. Let them. I won’t get it and no one in my family will get it. Don’t show up at my doorstep and try to force it on us either. It won’t end well. #nocovidvaccine #novaccine (2 January 2021).

Further, another reason for refusing the COVID-19 vaccine was justified through shared facts from unverified websites related to COVID-19 vaccine. The cited sources were linked to unverified websites like conspiracydailyupdate.com. For example, a Parler user claimed that:

A healthcare worker was diagnosed with Bell’s Palsy after administration of COVID-19 vaccine in Tennessee #sonotoneedles #wedonotconsent #novaccine #nocivilwar #nonewnormal #covid1984 #community #wakeup #nocovidvaccine (2 January 2021).

These parleys offer a genesis on the reasons behind Parler users’ refusal to get vaccinated. This also indicates that the role of health organizations, scientists, and public health experts is critical to address some of the concerns and doubts anti-vaxxers have through the use of social media. 

### 3.2. Side Effects of COVID-19 Vaccine

The second most prevalent theme in our findings was that users shared information about side effects from the COVID-19 vaccine. In particular, users shared information about possible adverse reactions such as getting Bell’s Palsy or even dying after receiving the vaccine. Several examples of such a parley are: 

Nurse Gets Bell’s Palsy (Paralyzed Face) After Taking Vaccine in Nashville, USA 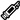
#covid19 #vaccine #vaxaware #vaxxed #coronavirus #notomandatoryvaccinations #nocovidvaccine #novax #wakeupcall #thegreatawakening #wakeupworld #usa #nurses #nurse #Tennessee #CoronaVirusUpdate #corona (29 November 2020).

Bell’s palsy isn’t the worst we’ve seen from this either cases of transverse myelitis, anaphylaxis, the appearance of H.I.V antibodies in a patient, sterilization possibilities for men and women, and worse death. Do not take this family! #sonotoneedles #wedonotconsent #novaccine #nocivilwar #nonewnormal #covid1984 #community #wakeup #nocovidvaccine (2 January 2021).

Tell that to the 100 s that have died globally, are sick in hospital, have Bell’s palsy or suffered mental damage. The lengths Canada is going to fool the population is far more terrifying than the virus. #trudeaumustgo #arrestdrtam #canada #vaccine #nocovidvaccine (9 January 2021). 

With the confluence of information on the platform, it is difficult to filter and separate factually grounded guidance from misinformation on side effects of the vaccine, potentially influencing users’ decision to vaccinate [25]. These parley examples demonstrate that users echo one another’s opinions on the platform on vaccine safety and efficacy. Further, Parler users warn of other side effects such as sterilization and even death. These parleys also induce fear among the users by stating adverse health effects of the vaccine that would heighten vaccine hesitancy.

### 3.3. COVID-19 Vaccine as a Population Control Mechanism 

Our data suggest that one of the most popular conspiracy theories included the use of COVID-19 vaccine to control the population. Specifically, users commented that Bill Gates and Dr. Anthony Fauci had instigated measures (i.e., microchips and enzymes in the vaccine) to control the population through the administration of the COVID-19 vaccine. For example: 

Bill Gates, the tech giant, is pushing the vaccine and pretending to be some medical expert. Why would he do this? Can you say microchip? Population control? New world order? No thank you sir. #novaccinemandates #nocovidvaccine #nocoronavaccine #exposebillgates #billgates #microchip #microchipping #donttakethemicrochip #vaccinemicrochips #populationcontrol (31 December 2020). 

AN ENZYME CALLED LUCIFERASE IS WHAT MAKES BILL GATES IMPLANTABLE QUANTUM DOT MICRONEEDLE VACCINE DELIVERY SYSTEM WORK -Serial #Covid19 #CoronaVirus #NoCovidVaccine #CoronaVirusFraud #VaccineFraud #Pfizer #Moderna #ECHO (21 December 2020).

Noticeably, many users included the hashtag #echo, which encourages other users to share these posts about conspiracy theories. These conspiracy theories were shared through links, videos, and images attached to the parleys. For instance, one user included a purported leaked Pentagon video saying that the vaccine was used to modify and control human behavior. Links cited contain information from non-governmental sources or do not contain any sources. Previous research on COVID-19 conspiracy theories and social media show that responsibility attribution concerns the country or entity who are responsible for causing a pandemic [37]. However, our results show that Parler users mainly blame famous personalities such as Bill Gates and Dr. Fauci, and no other clear responsibility attribution is present in the parleys. Further, users claim that powerful individuals such as Bill Gates are behind the creation of vaccines to gain social, economic, and political control over the population.

### 3.4. Children Getting Vaccinated without Parental Consent

Another theme that was prevalent in the data showed that children were getting vaccinated without parental consent. For example, Parler users voiced concerns that children would be forced to receive the vaccine. Users who voiced their concerns about children getting vaccinated without parental consent mentioned similar reasons as found in the literature, specifically relating to vaccine side-effects. Such examples include: 

I will not take the plandemic vaccine. I have 3 (2 who will NOT receive it) children and a husband who also will not take it. However, my oldest son is going to the navy at the end of summer and may end up being forced to take it and that makes us both reconsider him going. Which would be an enormous loss to the Navy, the American people, and the U.S. military as a whole. For me, forcing the covid vaccine on the military is a 100% deal breaker, for him it’s about a 65% deal breaker. He’s young and ignorant still, but also knows, moms are always right #scamdemic #plandemic #covid #nocovidvaccine #arrestfauci #arrestbillgates (30 December 2020). 

While we weren’t paying attention, they passed a law saying they can vaccinate children without parent’s consent and hide it from the parents... So, if your kid is at school, they can coerce a vaccine, hide that from you, then if your child has an adverse effect, you pay, they have indemnity, and if your child just drops dead, they still don’t have to tell you that your child was vaccinated without your consent or knowledge... Why else would the Ohio governor stop contact tracing at school? The end game is clear... Sterilize children, kill the sick and elderly, and do it under the guise of vaxxiNATIONal security. #nocovidvaccine #nomandatoryvaccinations #justsaynotovaccines #makethisgoviral #idonotconsent #idonotcomply (1 January 2021).

Parler users include the term “plandemic” in the parleys which infers that governments and certain powerful individuals “planned” this health crisis to vaccinate children without parental consent as part of the new world order to control future populations. With preexisting concerns among parents’ refusal to have their children vaccinated, users on Parler capitalize on these fears to further amplify already existing misinformation and conspiracy theories. The users also perpetuate the notion that COVID-19 vaccine is administered through coercion or required for military personnel to continue their services. Further, using strategies to contain the virus such as contact tracing, some Parler users noted that school administrators and governors can do anything to children under the guise of COVID-19 as a national security threat to vaccinate children. Parler users also mention the exploitation of younger populations who do not have adequate knowledge about vaccines.

### 3.5. Comparison of COVID-19 with Other Health Issues

In terms of comparison of COVID-19 with other health issues, our data reveal that Parler users make COVID-19 comparisons from four different fronts: magnitude of COVID-19 with other pandemics such as H1N1 and Ebola; vaccine efficacy such as H1N1 and flu vaccine; risk of getting infected with the virus; and downplaying the magnitude of COVID-19 in comparison to number of deaths caused by flu and abortion each year. For example:

I’m not an anti-vaxxer either, but if you know me you know how sick I got from the H1N1 vaccine. I hadn’t gone to the doctor for it, I was there for a different reason and he asked if I wanted it, so I said sure. I spent the next week in bed violently ill and the next several years suffering side effects. There is a lot more to the story and the pain and sickness the vaccine caused me. Needless to say, I’m not getting a Covid shot. Never again. #nocovidvaccine #covidvaccine (27 November 2020). 

The missing flu riddle: ’Influenza has been renamed COVID,’ maverick epidemiologist says #Covid19 #CoronaVirus #NoCovidVaccine #CoronaVirusFraud #Vaccines #VaccineMandate #Infertility #VaccineFraud” (20 December 2020). 

The number-one cause of death globally in 2020, with a record 42.7 million....was unborn babies killed in the womb. As of 31 December 2020, there were 42.7 million MURDERS of innocent babies. #coronavirus in 2020 totaled 1.8 million. Please learn more how you can #covid19hoax #covidhoax #plandemic #nocovidvaccine” (3 January 2021).

These Parler users discuss other health issues and compare the death toll of these threats to COVID-19 deaths. Several users downplay the COVID-19 severity by equating it to influenza. This may be interpreted as a move to allay their fears about and the risk of contracting the virus. Comparing statistics of abortion with COVID-19 deaths may lead individuals to be desensitized on the severity of the virus, making it seem less deadly and a hoax. Based on these parleys, we contend that individuals who may not have decided to get vaccinated yet may be less scared of the risk posed by COVID-19.

Our textual analysis revealed that individuals use words such as “plandemic” and “covid19hoax,” which shows that Parler users believe in widespread conspiracy theories about the origin of the pandemic and misinformation on vaccine efficacy. This corpus found 3,313 unique word formations. The analysis revealed the most frequent words and hashtags in the corpus were nocovidvaccine (348); vaccine (264); covid (184); covid19 (157); and vaccines (128). Table 1 provides more information on the top 20 words used by the Parler users to voice their opinions and perspectives on the COVID-19 vaccines.

**Figure 1:**
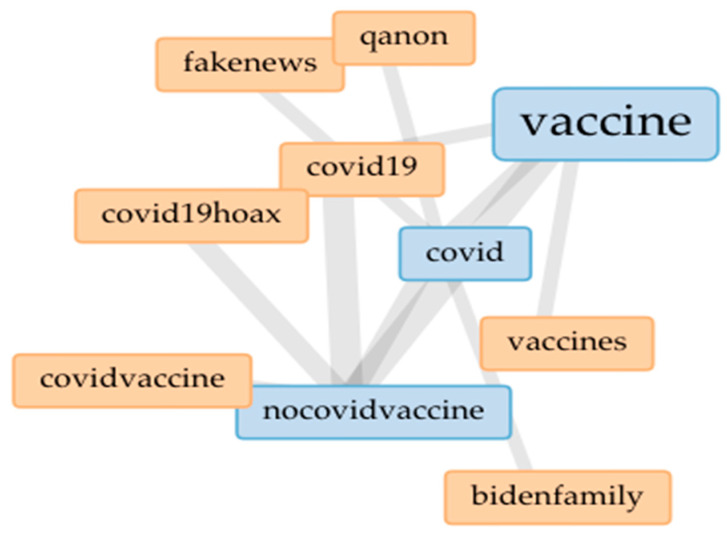
Collocates for #COVID19Vaccine and #NoCovidVaccine and on Parler.

**Figure 2:**
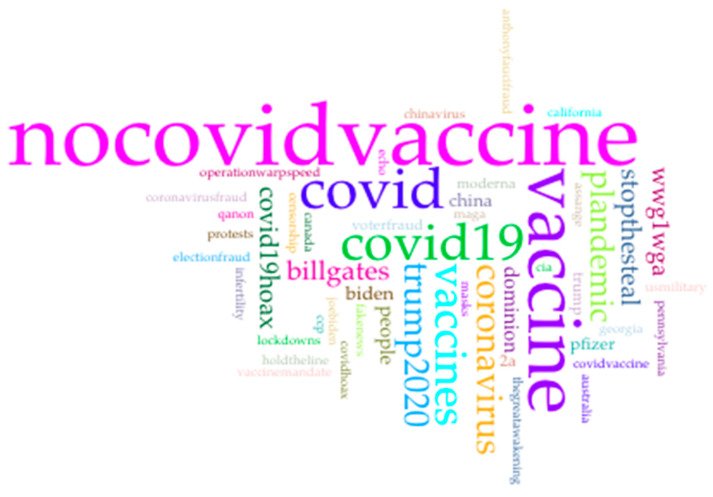
Word cloud depicting the most frequently used words by Parler users.

Figure 1 Shows the analysis of parleys and its “collocates” and word associations to provide more in-depth insights into the data. A word size indicates its frequency, and the lines show frequency of connections between words in parleys. Figure 1 also reveals that “nocovidvaccine” is associated with “covid19hoax”, which is a term that has been used by right-wing political leaders to discuss COVID-19. Figure 2, a word cloud, depicts the most frequently used hashtags such as #Nocovidvaccine, #Vaccine, #Covid, #Covid19, #Billgates, and #Trump2020. The bigger the word is depicted in the cloud, the more prevalent it is in the dataset. The word cloud findings resonate with our thematic analysis results. For instance, #Nocovidvaccine was the most frequently used hashtag in all the parleys.

## 4. Discussion and Conclusions

This study set out to examine online users’ discussions on the COVID-19 vaccine within the social media platform Parler. Based on the thematic analysis, the majority of parleys discussed reasons for vaccine refusal, COVID-19 vaccine side effects, population control through the COVID-19 vaccine, children getting vaccinated without parental consent, and comparison of other health issues with COVID-19. Our findings show that Parler users discuss several reasons for vaccine refusal and skepticism regarding the efficacy of the vaccine, refusal among healthcare workers to get vaccinated, and potential side effects of the vaccine. These findings are similar to previous research which reveal several reasons individuals choose to refuse vaccines [53]. The results from the first theme (reasons to refuse COVID-19 vaccines) show that users raise important and legitimate concerns about vaccine efficacy. Further, these findings suggest that individuals find medical authorities and vaccine development procedures are questionable. Due to medical mistrust, individuals voiced their opinion about vaccines on social media [54]. These questions about medical procedures are coming at a time that the world has experienced an unprecedented tumult, especially for health systems and scientists. Research shows that during crises individuals depend on media to get updated and accurate information [55]. To increase the public confidence on vaccines, public health experts and organizations should address individuals’ concerns about the vaccine efficacy. Some scholars have recommended that vaccine hesitant individuals need to be sensitized and included in civil dialogues online and offline [56]. It is pertinent to mention that fears, mistrust, and skepticism are somewhat justified among Parler users during this uncertain time. However, equally noteworthy is the fact that during the data collection period of the present study, COVID-19 vaccines were not yet administered to the public. Based on recent media reports, AstraZeneca (Vaxzevria) and Johnson & Johnson vaccines have been halted in several countries (i.e., Italy) and the American Food and Drug Administration (FDA) has called for an immediate precautionary suspension of the Johnson & Johnson vaccine to reevaluate their clinical trials of the vaccine. 

Secondly, Parler users identified and focused on side effects of the vaccine, specifically Bell’s Palsy. Medical research suggests that vaccinated people can have mild symptoms and side effects, however, there is minimal scientific evidence that shows a significant relationship between the COVID-19 vaccines and Bell’s Palsy [57]. Therefore, it is safe to say that Parler users are spreading misinformation that can ultimately lead individuals to become vaccine hesitant [58]. Recently, media reports showed that there have been concerns voiced among scientists regarding the side effects caused by the AstraZeneca and Johnson & Johnson vaccines [59]. However, only a few extreme side effect cases have been reported. Research shows that anti-vaxxers use different strategies or language including sharing information from pseudo-scientific accounts and websites to justify their viewpoints. Further, misinformation on COVID-19 vaccines can have dangerous consequences for those who are not anti-vaxxers, but it can affect their decision-making process after encountering these false claims. Another prevalent theme was population control through the COVID-19 vaccine in which powerful and prominent figures aim to control the population through vaccination. This theme is one of the novel findings of our study. Previous studies suggest that conspiracy theories influence people’s intentions to get vaccinated [37]. Additionally, children getting vaccinated without parental consent was another theme discussed among Parler users. The findings are interesting because children were not a priority group for many governments during the early phase of administering the COVID-19 vaccines. However, users were concerned without clear evidence that governments were forcing children to get vaccinated. Lastly, Parler users compare the COVID-19 pandemic with previous health epidemics including H1N1 and influenza. Further, users compare number of deaths caused by COVID-19 to that of abortions.

One of the major takeaways from the textual analysis findings is about the use of political terms and mention of political figures which allude to a political leader’s endorsement or lack of endorsement of the vaccine and the stance that the supporters should take on the issue through the use of hashtags such as #biden, #Trump, #Trump2020, #China, and #qanon. In terms of political ideologies, research shows that Trump voters are more concerned about vaccines than other politically affiliated Americans [60]. Putting our findings into perspective, our results align with preceding research that suggests belief in conspiracy theories is a major factor in generating and sustaining vaccine hesitancy [61]. 

Our results indicate that Parler, an example of a newer social media platform, is more prone to bolster vaccine hesitancy and conspiracy theories than older social media platforms such as Facebook and Twitter. This may be due to the unmoderated content policies of Parler as a social media platform. A recent study shows that unmoderated content on social media sites have larger impact than moderated content [62]. In response to concerns about COVID-19 vaccine hesitancy, Facebook, Google, and Twitter began moderating misinformation more actively related to vaccines [63]. 

The use of hyperlinks from pseudo-scientific accounts and websites was found to be prevalent among Parler users when it comes to suggesting health information sources to other users. The present study findings align with previous research that shows conservatives may consume untrustworthy news sources [64]. Some examples on Parler include humansarefree.com, conspiracydailyupdate.com, and welovetrump.com. The external links disseminated within the platform had lack of authenticity and reliability concerns. Prior research argues that links including foxnews.com, breitbart.com, par.pw, and bitchute.com were the top shared domains on Parler [8].

Parler offers new affordances for users to share unmoderated and unchecked claims. While other platforms such as Facebook, Instagram, and Twitter detect and filter out misinformation and add warning labels to the feed stating claims made by influential figures are disputed by official sources, social media platforms such as Parler, as of writing this paper, have yet to include algorithms designed for detecting false claims [65]. Specifically, during emergency situations and important events, social media sites become the main source for information seeking, however, the validation and credibility of online health information becomes more critical to evaluate [66]. Further, when moderation is present on social media platforms, dysfunctional conversations cannot easily survive filtration processes. Within any unmoderated network, this filter is less effective but social media platform policies are somewhat responsible for generating negative conversations and networks [62]. While this study shows legitimate concerns of the social media platforms in spreading misinformation, it is also important to highlight its potential as a tool to make individuals more aware of health issues such as vaccine effectiveness. 

The findings of this study show that the behavior of individuals on Parler regarding dissemination of the COVID-19 vaccine could have major implications for public health and the larger interest of society. Further, misinformation and conspiracy theories present on social media platforms like Parler are dangerous to not only decreasing vaccine acceptance, but also controlling future pandemics. In addition, it becomes more challenging for public health officials to inform the public about vaccine benefits. 

Many Parler users repeated similar hashtags, using words like “echo,” and joined to express and reinforce their ideologies with other like-minded people. Previous studies have shown that echo chambers have been under media scrutiny since they may lead to skewed assessments of factual information and lower the validity of information [19]. Studies further indicate that the homogeneity of the members within an echo chamber helps them to reinforce their biases, in this case COVID-19 vaccine refusal [24,67]. Based on our findings guided by the echo chamber conceptual framework, our study suggests that the Parler network is homogenous and the COVID-19 vaccine is discussed as a controversial issue. Some of the media reports critically examine that a majority of Parler users were conservative, Trump supporters, and have strong predispositions about conspiracy theories [68]. To conclude our discussion section, our findings suggest that individuals, specifically conservatives who already hold predispositions on vaccine efficacy, flock to Parler to create and participate in echo chambers.

To date, there have been considerable efforts in FDA-approved COVID-19 vaccine rollouts. However, it has been challenging for scientists to develop a vaccine that is 100% effective against COVID-19 due to the property of single-strand RNA. Additionally, within the short period of time, governments and scientists struggled with production, procurement, and making the vaccines largely available to the public [69]. At the time of writing this manuscript, more than 806 million people have been administered worldwide, equal to 10 doses for every 100 people [70].

## 5. Limitations and Future Studies

One of the limitations of this work is that we do not have demographic information of the Parler users, which hinders the generalizability of these findings. Second, Parler is a newly popular platform and, according to various news media reports, conservatives and right-wing audiences joined Parler right after the 2020 U.S. presidential election due to their reaction to political events and the COVID-19 pandemic [68]. Therefore, conducting an experiment to understand users’ behavior and vaccine intentions on Parler could provide a broader overview of our findings.

Another limitation is that we only analyzed textual data and excluded images, although we noted during the data collection process that a majority of users share images and videos to support their anti-vaccine beliefs on Parler. Future studies should evaluate the role of bots in conversations on social media platforms, especially pertaining to vaccine hesitancy. 

Our study is the first to examine the subject matter from a health perspective, with the other studies being centered on politics. Consequently, these studies will help policy makers to design effective communication strategies on social media. Local and state health departments also need to adopt and assess new social media channels to combat misinformation surrounding vaccines and empower citizens to take action and help society navigate the pandemic. According to CDC [71], one of the essential services for local health departments is to provide timely information at a large scale to audiences through social media platforms. Future studies also need to examine Rumble and Gab, among other newly launched platforms, to get a better sense of people’s opinions and perspectives through engagement with health topics. It would be helpful to conduct visual analysis of posts to explore the spread of misinformation online. Additionally, detecting the credibility of shared links in parleys through network analysis would be another area for future studies.

## Figures and Tables

**Table 1:** Top 20 most frequently used words in parleys.

No	Word	Frequency
1	nocovidvaccine	348
2	vaccine	264
3	covid	184
4	vaccines	128
5	coronavirus	109
6	trump2020	100
7	plandemic	95
8	billgates	86
9	stopthesteal	82
10	covid19hoax	78
11	dominion	67
12	people	67
13	biden	65
14	pfizer	64
15	china	63
16	trump	59
17	voterfraud	59
18	moderna	56
19	maga	49
20	echo	44
